# Differentiation of pulmonary solid nodules attached to the pleura detected by thin-section CT

**DOI:** 10.1186/s13244-023-01504-8

**Published:** 2023-09-12

**Authors:** Jin Jiang, Fa-jin Lv, Yang Tao, Bin-jie Fu, Wang-jia Li, Rui-yu Lin, Zhi-gang Chu

**Affiliations:** Department of Radiology, The First Affiliated Hospital of Chongqing Medical University, Chongqing, 400016 China

**Keywords:** Lung neoplasms, Diagnosis, Differential, Tomography, X-ray computed

## Abstract

**Background:**

Pulmonary solid pleura-attached nodules (SPANs) are not very commonly detected and thus not well studied and understood. This study aimed to identify the clinical and CT characteristics for differentiating benign and malignant SPANs.

**Results:**

From January 2017 to March 2023, a total of 295 patients with 300 SPANs (128 benign and 172 malignant) were retrospectively enrolled. Between benign and malignant SPANs, there were significant differences in patients’ age, smoking history, clinical symptoms, CT features, nodule-pleura interface, adjacent pleural change, peripheral concomitant lesions, and lymph node enlargement. Multivariate analysis revealed that smoking history (odds ratio [OR], 2.016; 95% confidence interval [CI], 1.037–3.919; *p* = 0.039), abutting the mediastinal pleura (OR, 3.325; 95% CI, 1.235–8.949; *p* = 0.017), nodule diameter (> 15.6 mm) (OR, 2.266; 95% CI, 1.161–4.423; *p* = 0.016), lobulation (OR, 8.922; 95% CI, 4.567–17.431; *p* < 0.001), narrow basement to pleura (OR, 6.035; 95% CI, 2.847–12.795; *p* < 0.001), and simultaneous hilar and mediastinal lymph nodule enlargement (OR, 4.971; 95% CI, 1.526–16.198; *p* = 0.008) were independent predictors of malignant SPANs, and the area under the curve (AUC) of this model was 0.890 (sensitivity, 82.0%, specificity, 77.3%) (*p* < 0.001).

**Conclusion:**

In patients with a smoking history, SPANs abutting the mediastinal pleura, having larger size (> 15.6 mm in diameter), lobulation, narrow basement, or simultaneous hilar and mediastinal lymph nodule enlargement are more likely to be malignant.

**Critical relevance statement:**

The benign and malignant SPANs have significant differences in clinical and CT features. Understanding the differences between benign and malignant SPANs is helpful for selecting the high-risk ones and avoiding unnecessary surgical resection.

**Key points:**

• The solid pleura-attached nodules (SPANs) are closely related to the pleura.

• Relationship between nodule and pleura and pleural changes are important for differentiating SPANs.

• Benign SPANs frequently have broad pleural thickening or embed in thickened pleura.

• Smoking history and lesions abutting the mediastinal pleura are indicators of malignant SPANs.

• Malignant SPANs usually have larger diameters, lobulation signs, narrow basements, and lymphadenopathy.

**Graphical Abstract:**

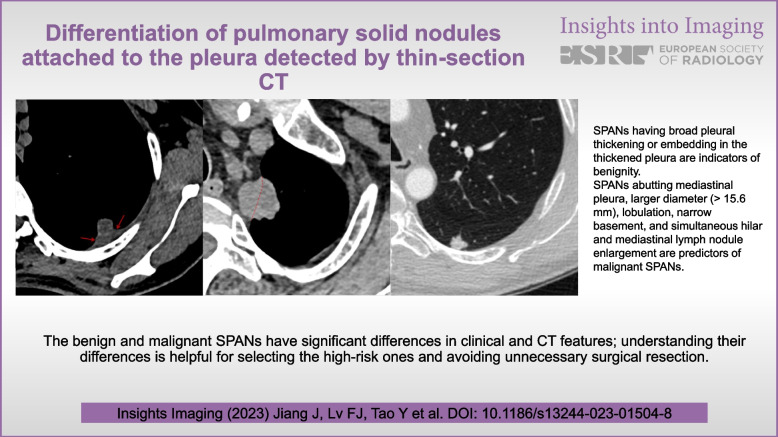

**Supplementary Information:**

The online version contains supplementary material available at 10.1186/s13244-023-01504-8.

## Introduction

Pulmonary nodule is a common lung abnormality, which has been commonly detected with the development of computed tomography (CT) [1]. The detected nodules can be divided into solid nodules (SNs) and subsolid nodules (SSNs). At present, the researches on the morphological characteristics of neoplastic and non-neoplastic SSNs have been relatively sufficient [2–8]. Additionally, the signs used to differentiate them are relatively consistent in different studies. Compared with SSNs, the etiology of SNs is more diverse, and they are frequently misdiagnosed on CT images. In view of the relatively higher growth rate and worse prognosis of malignant SNs [9, 10], it is necessary to further reveal the indicators for differentiating them.

On CT images, the diagnosis of SNs mainly depends on the display of their detailed manifestations [11]. Among the peripheral ones, some are abutting the pleura and thus a part of CT features are not significant, such as the pleural indentation sign, which makes the solid pleura-attached nodules (SPANs) more difficult to be differentiated by using the traditional morphological features. Additionally, visceral pleural invasion (VPI) is more likely to develop in malignant SPANs, and the presence of VPI is associated with a higher incidence of lymph node metastasis, making sublobar resection, with its limited lymph node sampling and dissection, a less effective treatment approach [12, 13]. Therefore, it should pay more attention to differentiating SPANs with the aim of early detecting the malignant ones.

Previous studies have demonstrated that solid noncalcified nodules attached to the costal pleura less than 10.0 mm with lentiform, oval, semicircular, or triangular shapes and smooth margins were benign at low-dose CT screening for lung cancer [14, 15]. However, they excluded nodules attached to the mediastinal and diaphragmatic pleura. In other studies, they believed that nodules abutting the pleura and SPANs with broad basements to the pleura were indicators of benign lesions [16–18]. However, they did not further compare the benign and malignant nodules because of the small number of samples [14–18]. Additionally, some studies only focused on revealing CT features suggestive of VPI in malignant SPANs [19–23]. Therefore, the characteristics used to identify SPANs as benign or malignant are still relatively lacking.

Since SPANs are intimately related to the pleura, the relationship between nodule and pleura may provide additional information for differential diagnosis. However, their relationship was not evaluated adequately in previous studies, and there is no study to verify its value in distinguishing them. Thus, the studies regarding the differential diagnosis of SPANs are insufficient. In this study, the clinical and CT characteristics of benign and malignant SPANs were evaluated thoroughly with the aim of determining the key indicators for predicting malignancy.

## Materials and methods

This retrospective study was approved by the Institutional Review Board of the First Affiliated Hospital of Chongqing Medical University, and the requirement for informed consent was waived due to the retrospective nature of this study.

### Patients

**Fig. 1:**
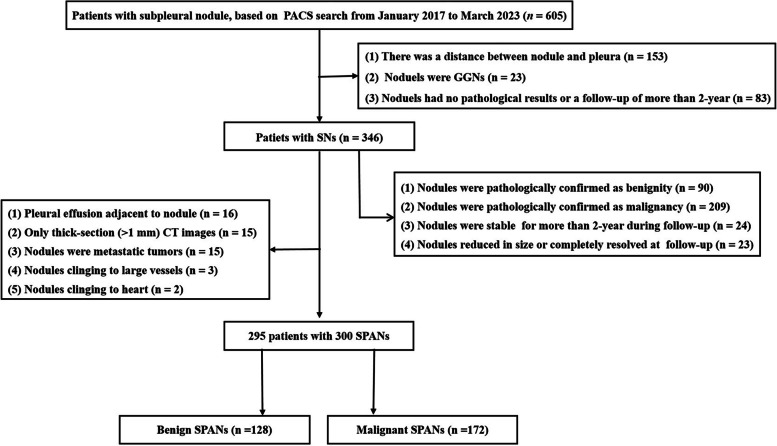
Flowchart of the study population. PACS, picture archiving and communication system; SNs, solid nodules; GGN, ground-glass nodule; SPANs, solid pleura-attached nodules

The picture archiving and communication system (Carestream Vue PACS) was searched for patients who had undergone CT chest examinations and whose radiological diagnosis or radiological manifestations included subpleural nodules from January 2017 to March 2023. The nodules with pathological or clinical diagnosis were used for further study. In this study, all of the malignant nodules were confirmed by pathological examination. Nodules were seen as benignity when either (a) the nodules were confirmed as benign lesions by pathological examination, (b) the nodules were stable for more than 2 years during follow-up, or (c) the nodules significantly reduced in size or completely resolved at subsequent follow-up. The enrolled patients required the following conditions to be satisfied: (1) the lesions were solid nodules (diameter ≤ 3 cm) on CT images, (2) there was no distance between nodule and adjacent pleura, (3) the lesions were abutting the mediastinal pleura or costal pleura, and (4) patients with complete clinical and CT data. The excluded patients required the following conditions to be satisfied: (1) the nodules had significant calcification, (2) absence of thin-section CT images (≤ 1.5 mm), (3) presence of artifacts on CT images affecting evaluation, (4) presence of pleural effusion around the nodules, (5) nodules abutting the pleura which was adjacent to the large vessels or heart, and (6) nodules were confirmed as metastatic tumors. The patients’ selection procedure is shown in Fig. 1.

### CT examinations

Among the 295 patients, 118 (40%), 111 (37.6%), 46 (15.6%), 14 (4.7), and 6 (2.0%) were examined using SOMATOM Perspective (Siemens Healthineers, Erlangen, Germany), Discovery CT750 HD (GE Healthcare, Milwaukee, WI, USA), SOMATOM Definition Flash (Siemens Healthineers, Erlangen, Germany), SOMATOM Force (Siemens Healthineers, Erlangen, Germany), and Aquilion ONE pureViSION (Canon Medical System, Japan), respectively. All patients were placed in a supine position with raised upper limbs and were asked to hold their breath after deep inspiration for better exposure. The scan range was from the thoracic entrance to the costophrenic angle. The following were the scan parameters: tube voltage, 110–130 kVp; tube current time, 50–140 mA (using automatic current modulation technology); scanning slice thickness, 5 mm; rotation time, 0.5 s; pitch, 1–1.1; collimation, 0.6 or 0.625 mm; reconstruction slice thickness and interval, 0.625 or 1 mm; and matrix, 512 × 512. All patients underwent plain CT scan, and 103 (34.9%) of them (40 with benign lesions and 63 with malignant lesions) underwent contrast-enhanced CT scan with a total of 80–100 mL of nonionic iodinated contrast material (iopamidol, 320 mg/mL; Shanghai Bracco Sine Pharmaceutical Co., Ltd., China) at an injection rate of 3.0 mL/s, followed by 50 mL of saline solution via a power injector. Images were obtained with mediastinal (width, 350–400 HU; level, 20–40 HU) and lung (width, 1200–1600 HU; level, − 500 to − 700 HU) window settings.

### Image analysis

The Electronic Medical Record System (Winning Health, China) was used to record patients’ clinical data. Clinical data, including the patient’s age, sex, history of malignant tumor, smoking history, drinking history, clinical symptoms (cough, expectoration, phlegm with blood, and chest pain), and other lung diseases were recorded and evaluated. Lung disease includes chronic obstructive pulmonary disease, bronchial asthma, pulmonary tuberculosis, and pneumonia.

A picture archiving and communication system workstation (Carestream Vue PACS) with lung window settings (width, 1200–1600 HU; level, − 500 to − 700 HU) and mediastinal window setting (width, 350–400 HU; level, 20–40 HU) was used for CT data analysis. CT data from all patients were independently evaluated by two radiologists (Y.T. and Z.G.C.) with more than 6 years of experience in chest CT interpretation, who were blinded to the pathological results of nodules, and the discrepancy between two radiologists was resolved by consensus.

**Fig. 2:**
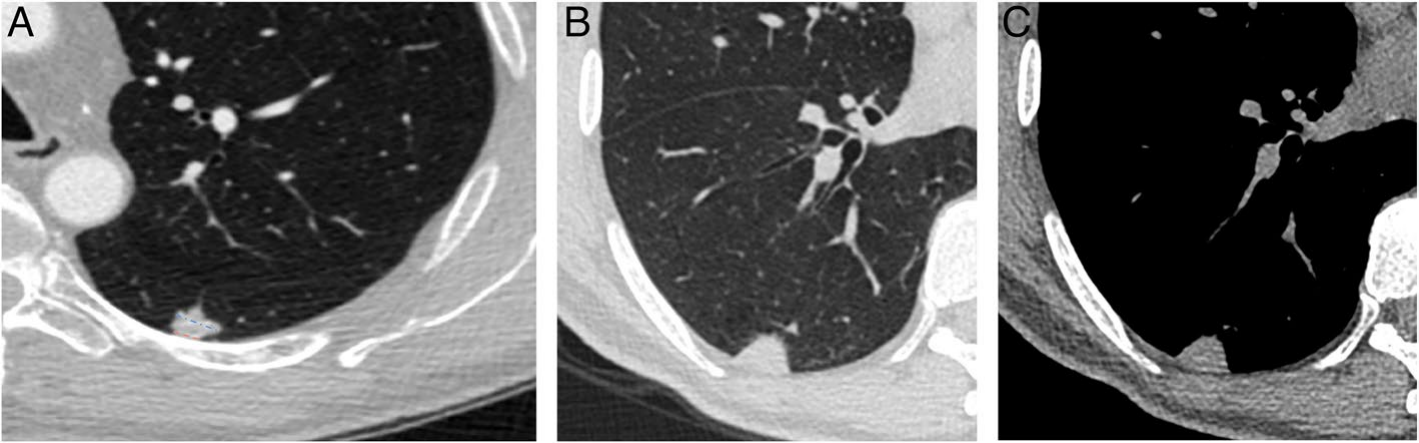
SPAN with narrow or broad basement and type I of nodule-pleura relationship. **a** A 59-year-old man with left lower lobe lobulated SPAN which is confirmed as invasive adenocarcinoma. On the axial CT image with a maximum section of the nodule, R-length is defined as the ratio of the length of the nodule-pleura interface (red dashed line) to nodule size (blue dashed line). Its R-length is 0.36, which indicates a narrow basement. The nodule-pleura relationship is type I (nodule clinging to the normal pleura without extrapleural fat infiltration). **b**, **c** A 49-year-old man with right lower lobe SPAN which is confirmed as a benign lesion during follow-up. On the axial CT image with a maximum section of the nodule, its R-length is 1, which indicates a broad basement, and the nodule-pleura relationship is also type I

**Fig. 3:**
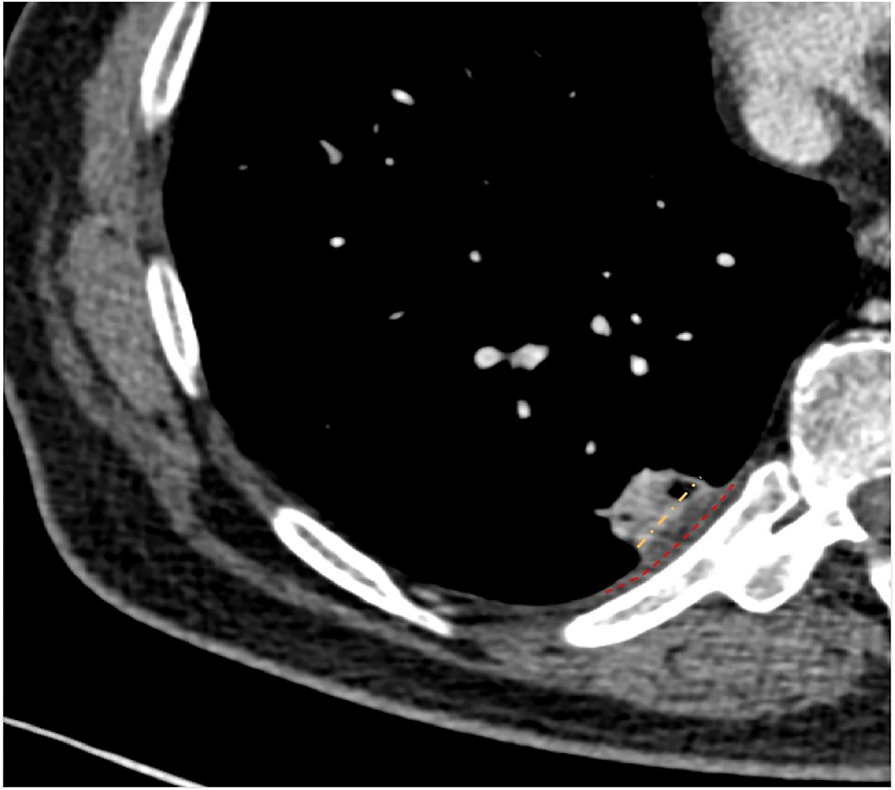
SPAN with pleural thickening and type II of nodule-pleura relationship. A 53-year-old man with right lower lobe SPAN which is confirmed as an inflammatory lesion. On the axial enhanced CT image, it is found that there is a visible increase in pleural thickness (red dashed line) beyond the normal pleural appearance adjacent to the nodules (pleural thickening). R-thickening is defined as the ratio of the length of adjacent thickened pleura (red dashed line) to nodule diameter (yellow dashed line). Its R-thickening is 1.39. The nodule-pleura relationship is type II (nodule clinging to the thickened pleura without pleural infiltration)

**Fig. 4:**
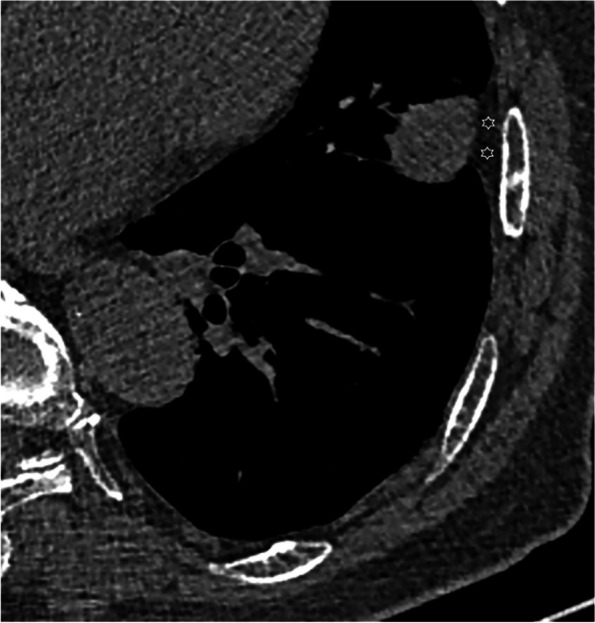
SPAN with extrapleural fat thickening. A 76-year-old man with left upper lobe SPAN which is confirmed as invasive adenocarcinoma. On the axial CT image, significantly thickened extrapleural fat (asterisks) protrudes into the lung field

**Fig. 5:**
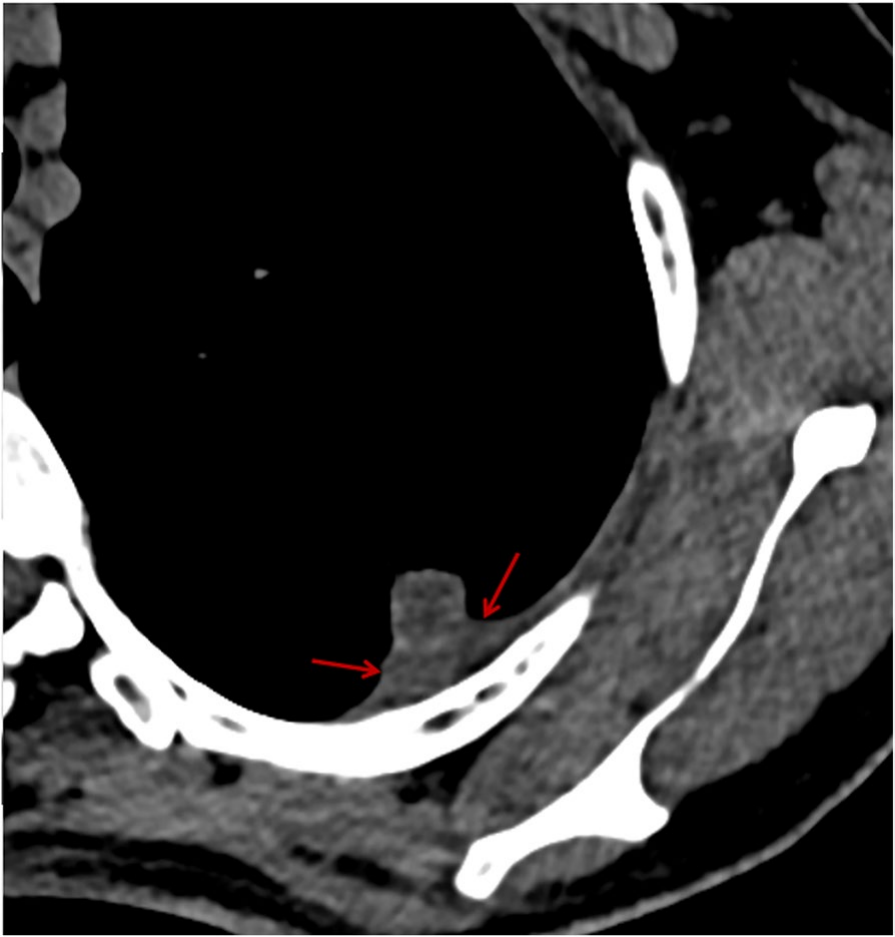
SPAN with type III of nodule-pleura relationship. A 48-year-old woman with left upper lobe SPAN which is confirmed as a benign lesion during follow-up. On the axial CT image, a part of the nodule with slightly high density embeds in the significantly thickened pleura (red arrows)

**Fig. 6:**
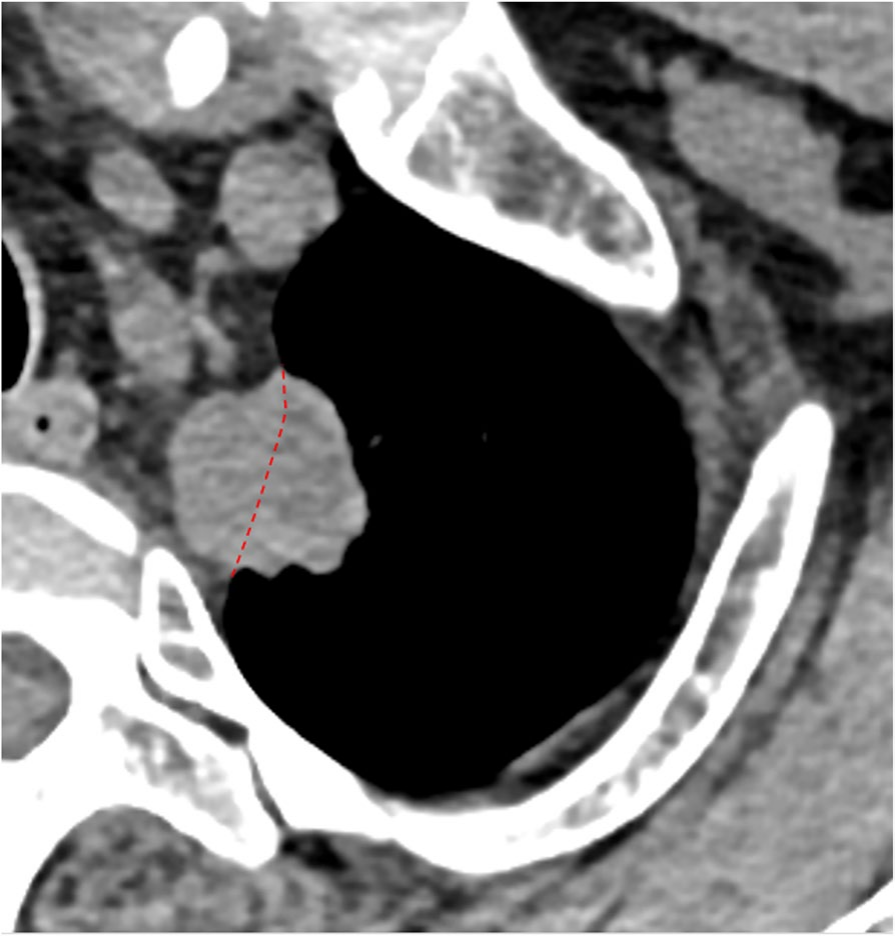
SPAN with type IV of nodule-pleura relationship. A 44-year-old man with left upper lobe SPAN which is confirmed as an invasive adenocarcinoma. On the axial CT image, the lobulated nodule crosses the potential pleural line (dashed line) and extends into extrapleural fat

The following CT features of nodules were analyzed based on the plain and enhanced CT images: size (the mean of the longest diameter and the perpendicular diameter on axial CT images), distribution (upper, middle, or lower lobe), location (abutting the mediastinal pleura or costal pleura), shape (oval, round, or irregular), lobulation, spiculation, R-length (Fig. 2), adjacent pleural thickening (Fig. 3), R-thickening (Fig. 3), extrapleural fat thickening (Fig. 4), the relationship between nodule and pleura, concomitant lesions in peripheral lung fields, intrathoracic lymph node enlargement, and CT value on plain and enhancement value. Lobulation was defined as an abrupt bulging of the contour of the lesion [24]. Spiculation was defined as linear strands that extended from the nodule surface into the lung parenchyma without reaching a pleural surface [25]. R-length was measured on transverse, coronal, or sagittal CT images with a maximum section of the nodule. R-length ≥ 1 indicated a broad basement, or it was narrow basement. R-thickening ≥ 1 indicated broad pleural thickening, or it was narrow pleural thickening. The relationship between nodule and the pleura was classified into four types (I, nodule abutting the normal pleura; II, nodule abutting the thickened pleura; III, nodule embedding in thickened pleura; IV, nodule infiltrating extrapleural fat) (Figs. 2, 3, 5, and 6). Concomitant lesions in peripheral lung fields of nodules included scattered patchy consolidation or ground glass opacity, nodules, or fibrosis. Mediastinal and hilar lymph node enlargement was defined as those with a diameter of more than 1 cm in the short axis on chest CT scans [26]. The enhancement value was the difference between the peak CT value on a contrast-enhanced CT scan and the CT value on a plain CT scan.

### Statistical analysis

Clinical data and various CT features were statistically analyzed for each patient. Continuous variables were expressed as mean ± standard deviation, and categorical variables were expressed as number and percentage. The Mann–Whitney *U*-test was used for patients’ age, nodule size, plain CT value, degree of enhancement, and R-thickening, and the Pearson chi-square test was used for sex, history of malignant tumor, smoking history, drinking history, clinical symptoms, number of cases with other lung diseases, lesion location, and frequencies of different CT features. Eventually, we included all variables in the logistic regression model to obtain independent predictors of malignant SPANs. If *p* < 0.05, this factor was considered a statistically significant difference.

## Results

### Patients’ clinical characteristics

Among the 128 benign nodules, 31 (24.2%) were stable for more than 2 years, 21 (16.4%) were completely absorbed during follow-up, and 53 (41.4%), 14 (10.9%), 7 (5.4%), 2 (1.6%) were pathologically confirmed as nonspecific inflammatory lesions, fungal infection, tuberculosis (TB), and hamartoma, respectively. The 172 malignant nodules included 121 (70.3%) adenocarcinomas, 40 (23.3%) squamous cell carcinomas, 8 (4.7%) small cell lung cancers, 2 (1.2%) adenosquamous carcinomas, and 1 (0.6%) sarcomatoid carcinomas. The patients’ clinical characteristics are summarized in Table 1. Compared with patients with benign SPANs, cases were older (*p* < 0.001), and smokers (*p* < 0.001) and individuals with clinical symptoms (*p* = 0.027) were more common in those with malignant ones.

**Table 1:** Patients’ clinical characteristics

Characteristics	Patients with benign SPANs (*n* = 125)	Patients with malignant SPANs (*n* = 170)	*p*-value
Age (years)	58.78 ± 13.94	64.23 ± 10.78	< 0.001
Sex			0.099
Female	49 (39.2)	51 (30.0)	
Male	76 (60.8)	119 (70.0)	
History of malignant tumor			0.057
Yes	31 (24.8)	27 (15.9)	
No	94 (75.2)	143 (84.1)	
Smoking history			< 0.001
Yes	47 (37.6)	104 (61.2)	
No	78 (62.4)	66 (38.8)	
Drinking story			0.083
Yes	35 (28.0)	64 (37.6)	
No	90 (72.0)	106 (62.4)	
Cases with clinical symptoms			0.027
Yes	47 (37.6)	86 (50.6)	
No	78 (62.4)	84 (49.4)	
Cases with other lung disease			0.604
Yes	13 (10.4)	21 (12.4)	
No	112 (89.6)	149 (87.6)	

*Notes*: Data are expressed as number (percentage) or mean ± standard deviation

### CT features of SPANs

The CT features of SPANs are summarized in Table 2. The malignant SPANs were larger than the benign ones, and the optimal cutoff value of diameter for distinguishing them obtained by using the receiver operating characteristic (ROC) curve was 15.6 mm. Compared with benign SPANs, more malignant ones abutted the mediastinal pleura and had lobulation, spiculation, narrow basement to the pleura, narrow pleural thickening, extrapleural infiltration, and simultaneous hilar and mediastinal lymph nodule enlargement (each *p* < 0.05).

**Table 2:** CT characteristics of the benign and malignant nodules

Characteristics	Benign SPANs (*n* = 128)	Malignant SPANs (*n* = 172)	*p*-value
Distribution			0.053
Upper lobe	54 (42.2)	92 (53.5)	
Middle and lower lobes	74 (57.8)	80 (46.5)	
Abutting the mediastinal pleura	10 (7.8)	43 (25.0)	< 0.001
Size (mm)	14.55 ± 6.84	19.18 ± 6.07	< 0.001
Shape (irregular)	8 (6.3)	9 (5.2)	0.706
Lobulation	23 (18.0)	128 (74.4)	< 0.001
Spiculation	25 (19.5)	77 (44.8)	< 0.001
Narrow basement	65 (50.8)	137 (79.7)	< 0.001
Adjacent pleural change			0.229
No	101 (78.9)	144 (83.7)	
Pleural thickening	27 (21.1)	28 (16.3)	
R-thickening	1.31 ± 0.68	0.75 ± 0.27	< 0.001*
Extrapleural fat thickening	14 (10.9)	24 (14.0)	0.437
Relationship between nodule and pleura			< 0.001
Type I	99 (77.3)	126 (73.3)	0.419
Type II	12 (9.4)	24 (13.4)	0.145
Type III	15 (11.7)	3 (1.7)	< 0.001
Type IV	2 (1.6)	20 (11.6)	0.001
Concomitant lesions in peripheral lung fields (yes)	51 (39.8)	43 (25.0)	0.006
Lymph node enlargement			0.002
No	112 (87.5)	122 (70.9)	0.001
Hilar or mediastinal lymph nodule enlargement	9 (7.0)	24 (14.0)	0.058
Simultaneous hilar and mediastinal lymph nodule enlargement	7 (5.5)	26 (15.1)	0.008
Plain CT value (HU)	37.17 ± 30.0	33.06 ± 17.67	0.802
△CT value (HU)	45.85 ± 23.50	42.05 ± 19.17	0.463*

Data are expressed as number (percentage) or mean ± standard deviation

^*^Only the patients with this data were compared

### Logistic regression analysis for benign and malignant PSNs

Table 3 shows the clinical and CT features which can independently distinguish benign and malignant SPANs via logistic regression. Smoking history, abutting the mediastinal pleura, nodule diameter > 15.6 mm, lobulation, narrow basement to the pleura, and simultaneous hilar and mediastinal lymph nodule enlargement were revealed as independent indicators for predicting malignant SPANs (each *p* < 0.05). The sensitivity, specificity, accuracy, and area under the curve (AUC) (Fig. S1) for this model were 82.0%, 77.3%, 80.0%, and 0.890 (95% CI: 0.853–0.927) (*p* < 0.001), respectively.

**Table 3:** Multivariate logistic regression for predicting malignant nodules

Variable	Odds ratio (95% CI)	*p*-value
Smoking history		0.039
No	1	
Yes	2.016 (1.037, 3.919)	
Abutting the mediastinal pleura		0.017
No	1	
Yes	3.325 (1.235, 8.949)	
Size (mm)		0.016
< 15.6	1	
> 15.6	2.266 (1.161, 4.423)	
Lobulation		< 0.001
No	1	
Yes	8.922 (4.567, 17.431)	
Attachment to the pleura		< 0.001
Broad	1	
Narrow	6.035 (2.847, 12.795)	
Relationship between nodule and pleura		0.002
Type I	1	
Type III	0.069 (0.013, 0.368)	
Lymph node enlargement		0.008
No	1	
Simultaneous hilar and mediastinal lymph nodule enlargement	4.971 (1.526, 16.198)	

## Discussion

SPANs as a special kind of pulmonary nodules, they are not very commonly detected and thus not well studied and understood. The benign and malignant SPANs present great similarities in their clinical and CT features, but they still have some differences which are particularly important for further differential diagnosis. According to the present results, it was found that smoking history, larger diameter (> 15.6 mm), lobulation, abutting the mediastinal pleura, narrow basement to the pleura, and simultaneous hilar and mediastinal lymph nodule enlargement were independent indicators for predicting malignant SPANs. Thus, patients’ clinical characteristics, morphological features of nodules, and the relationship between nodule and pleura should be comprehensively evaluated in the differentiation of SPANs.

It was revealed that the clinical risk factors indicating lung cancer include age and smoking history because immunity generally decreases with age, but total exposure to carcinogens increases with increasing years of smoking [27, 28]. In this study, older patients and smokers were more common in those with malignant lesions, which was identical to the previous results [29]. Though more patients with malignant SPANs in this study had clinical symptoms, such as cough and expectoration, which were not seen as the results caused by tumors but may be related to that the older individuals had greater susceptibility to chronic lung disease, whether patients have symptoms or not cannot be seen as an indicator of malignant or benign lesions.

Previous studies have reported that malignant nodules are more likely to be located in the upper lobes [16, 30]. However, the present results suggested that there was no significant tendency in the distribution of benign and malignant SPANs. In contrast, though SPANs frequently abutted the costal pleura because the latter was more extensive, the proportion of cases abutting the mediastinal pleura in malignant ones was significantly higher than that in benign ones. Similarly, mediastinal pleural involvement is more important than costal pleural involvement for predicting malignant pleural mesothelioma [31]. This new finding suggests that the location of nodule relative to pleura is more useful for differentiating SPANs than the distribution in different lobes. Thus, the SNs abutting the mediastinal pleura should be firstly excluded as malignant lesions.

The larger the size and the more irregular the shape, the greater the likelihood of malignancy [14]. In this study, between benign and malignant nodules, there were significant differences in size but not in shape. The optimal cutoff value of diameter for distinguishing malignant nodules from benign ones was > 15.6 mm, which was similar to that of transient nodules [32]. In addition, lobulation and spiculation were all more common in malignant SPANs than in benign ones. These findings suggested that the benign and malignant SPANs shared similar characteristics of all peripheral SNs [33]. Though these two features were useful for differentiating SPANs, none of them was specific, and the spiculation was not an independent indicator for predicting malignant ones. In clinical practice, they could not be used solely, especially the latter, but should be combined with others for differential diagnosis.

Though the benign and malignant SNs in this study all adhere to the pleura, they showed some difference in the nodule-pleura interface. The narrow basement was more common in malignant SPANs because the tumors usually grew at different rates in all directions and involved the pleura only when they were large enough, which was identical to previous studies [16, 17]. In malignant SPANs, the higher contact length-to-size ratio, the greater the likelihood of pleural invasion and the worse the prognosis [19, 20, 23]. So, it is necessary to effectively differentiate the SPANs with broad basements.

Pathologically, pleural thickening was due to fibroblastic proliferation, prominent elastic reduplication, inflammatory infiltrates, and fibrosis [12]. Lesions with different pathological natures may cause different types of pleural thickening [34, 35]. In this study, the proportion of cases with pleural thickening in benign and malignant SPANs was similar, while the thickened pleura was more extensive in the former. This difference may be due to the reason that the inflammatory process as the common reason for benign nodules usually resulted in significant pleural swelling while there was no significant pleural involvement until the adjacent pleura was invaded [17, 22]. Thus, the degree of pleural thickening adjacent to lesions is more important than the occurrence of pleural thickening in differentiation. Additionally, extrapleural infiltration was more common in malignant SPANs, while nodules embedding in the thickened pleura was seen as characteristic of benign ones. These findings were not reported in previous studies. However, extrapleural infiltration was not specific to malignant nodules, which also presented in fungal infections or tuberculosis, as they were pathologically granulomatous inflammation [36]. On enhanced CT images, the contrast between nodules with significant enhancement and pleura without significant enhancement was more obvious; therefore, it would be best to differentiate SPANs on enhanced CT images.

Peripheral sporadic patches, nodules, or fibrosis were common for inflammatory lesions because of the infiltration of massive inflammatory cells and fibrous tissue proliferation, which could help distinguish them from lung cancers [18]. In this study, the ground glass opacity and fibrosis in peripheral lung fields of nodules were more common in benign lesions, which was consistent with previous findings [17, 18]. Hence, the occurrence of concomitant lesions in peripheral lung fields may provide additional information for differential diagnosis.

Lymph node enlargement was generally found in neoplastic lesions or inflammatory ones [37]. Previous studies have shown that hilar lymph node enlargement usually occurred in focal inflammatory lesions, while metastasis of lung cancer was more likely to cause simultaneous hilar and mediastinal lymph node enlargement [17]. In the present study, simultaneous hilar and mediastinal lymph node enlargement was also more common in patients with malignant SPANs. Thus, lung cancer should be first considered, and further examination should be taken to confirm this possibility once this indicator is positive.

Our study had some limitations. First, the sample in this single-center study was relatively small. Second, not all the patients had enhanced CT data; thus, some features partly rely on enhanced CT images may not adequately evaluated. For these patients, some of the CT features of lesions were evaluated by adjusting the window width and window level. Third, the evaluation of extrapleural infiltration was subjective, which was not confirmed by pathological examination due to the deficiency of retrospective studies. Therefore, the current results should be further verified in clinical practice.

## Conclusion

SPANs as a special kind of peripheral pulmonary nodules, in combination with patients’ clinical characteristics, their CT features, changes of the adjacent pleura, and concurrent lesions, are necessary for their differential diagnosis. SPANs abutting the mediastinal pleura, having larger size (> 15.6 mm in diameter), lobulation, narrow basement to the pleura, or simultaneous hilar and mediastinal lymph nodule enlargement are more likely to be malignant. Realizing the diversities in clinical and CT features between benign and malignant SPANs is helpful for selecting the high-risk ones and avoiding unnecessary surgical resection.

### Supplementary Information

**Additional file 1: Fig. S1.** Receiver operating characteristic curves of the malignant SPANs predictive model established by the independent clinical and CT characteristics mentioned above.

## Data Availability

The datasets generated and/or analyzed during the current study are not publicly available because the cases are from the Picture Archiving and Communicating System of our Hospital but are available from the corresponding author upon reasonable request.

## Abbreviations

AUC: Area under the curve
CT: Computed tomography
OR: Odds ratio
ROC: Receiver operating characteristic
SNs: Solid nodules
SPANs: Solid pleura-attached nodules
SSNs: Subsolid nodules
TB: Tuberculosis
VPI: Visceral pleural invasion
