# Supplementary material for: Differentiation of pulmonary solid nodules attached to the pleura detected by thin-section CT
Source: Insights Imaging. 2023 Sep 12;14:146. doi: 10.1186/s13244-023-01504-8 (PMC10495292; doi:10.1186/s13244-023-01504-8)
Supplement: Supplementary file 1 — Additional file 1: Fig. S1. Receiver operating characteristic curves of the malignant SPANs predictive model established by the independent clinical and CT characteristics mentioned above. [file 13244_2023_1504_MOESM1_ESM.pdf]

Differentiation of pulmonary solid nodules attached to the pleura detected by thin-section CT

ELECTRONIC SUPPLEMENTARY MATERIAL

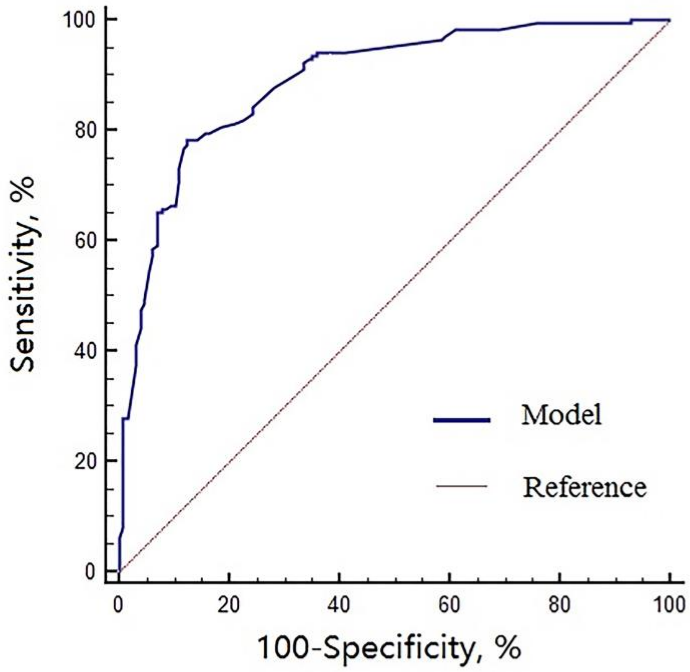

Fig. S1.

Receiver operating characteristic curves of the malignant SPANs predictive model established by the independent clinical and CT characteristics mentioned above.
